# Evaluation of stress distribution in and around dental implants using three different implant–abutment interfaces with platform-switched and non-platform-switched abutments: A three-dimensional finite element analysis

**DOI:** 10.34172/joddd.2023.40723

**Published:** 2023-12-30

**Authors:** Dipika Mitra, Prachi Gurav, Silvia Rodrigues, Bela Khobragade, Amruta Mahajan

**Affiliations:** ^1^Department of Periodontology, TPCT’s Terna Dental College & Hospital, Navi Mumbai, Maharashtra, India; ^2^Department of Periodontics and Implantology Terna Dental College & Hospital, Navi Mumbai, Maharashtra, India

**Keywords:** Dental implants, Dental implant abutment design, Dental stress analysis, Finite element analysis

## Abstract

**Background.:**

A key factor for the success or failure of an implant is how the stresses are transferred to the surrounding bone. The implant‒abutment connection (IAC) is paramount for implant success. The purpose of this finite element analysis (FEA) study was to evaluate the stress distribution in and around three different implant‒abutment interfaces with platform-switched and platform-matched abutments using the finite element method (FEM).

**Methods.:**

Three distinct types of IAC were selected: tri-channel internal connection, conical connection, and internal hex connection. Six models were generated, three in platform-switched and three in non-platform-switched configuration. Computer-Aided Three-Dimensional Interactive Application (CATIA) V5 R20 software was used to generate virtual models of the implants and the mandible. The models were transferred to Analysis of Systems (ANSYS) 15.0 software, in which the models were meshed and underwent FEA.

**Results.:**

On the crestal bone, the highest von Mises stresses in platform-switched abutments were noticed in the internal hex implant‒abutment system (370 MPa), followed by the tri-channel implant‒abutment system (190 MPa) and conical implant‒abutment system (110 MPa). On the implant and the abutment screw, the highest von Mises stresses were observed in the internal hex implant‒abutment system, followed by the conical implant abutment system and tri-channel implant‒abutment system. Platform-switched implants had a more favorable stress distribution on crestal bone.

**Conclusion.:**

Within the constraints of the current study, the internal hex connection exhibited the highest stress. In contrast, the conical abutment connection with platform switching configuration had more favorable stress distribution in crestal bone than other implant abutment systems.

## Introduction

 Implants were introduced in the 1970s for the rehabilitation of patients who were completely edentulous.^1,2^ Since then, there has been a greater awareness of, and consequent demand for, this type of therapy. How stresses are transmitted to the surrounding crestal bone is a crucial aspect of the success of a dental implant. The type of loading, the bone‒implant contact, the dimensions of the implants, and the quality and quantity of the bone all influence load transmission from implants to the surrounding bone.^3^

 An implant‒abutment interface is the level at which the abutment connects to the implant body and is also called the implant‒abutment connection (IAC) or the implant‒abutment junction (IAJ).^4^ Research has shown that the type of IAC can affect the stresses generated in peri-implant bone, resulting in crestal bone loss.^5-7^ There are several types of IAC, such as the external hex and internal hex connections. Commonly used internal connections include hexagons, octagons, and tri-channels as part of their internal geometry.^8^

 Research has shown that platform-matched implants have demonstrated early bone loss in the peri-implant region.^5^ Therefore, to preserve the crestal bone, a stable IAC should be obtained by reducing the diameter of the abutment. The situation in which the abutment is narrower than the implant at the connection is termed platform switching. According to several studies, platform switching reduces crestal bone loss around the implant while allowing for a greater volume of soft tissue at the IAC to aid in soft tissue esthetics.^9,10^

 Weinstein et al^11^ in 1976 introduced finite element analysis (FEA) in implantology. In finite element method (FEM), virtual models are created to simulate complex structures, and the stress distribution is tested on these models. Thus, the inherent shortcomings of in vivo and in vitro methods for experimentally analyzing and evaluating the biomechanical behavior of the bone, implant, and prosthetic component is easily overcome by FEM. It enables researchers to apply loads in different directions and calculate the stress levels on the oral structures and implants.^3^

 Hence, this study evaluated the stress distribution in and around three implant‒abutment interfaces with platform-switched and platform-matched abutments using FEA.

## Methods

###  Generation of models

**Figure 1:**
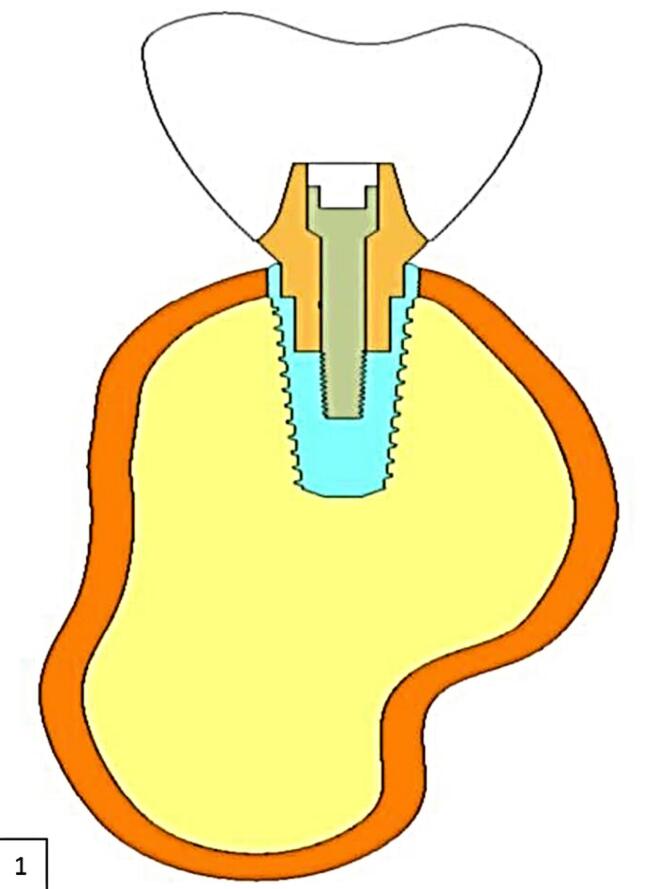
Two-dimensional representation of the CAD model of the mandible with implant

 A finite element (FE) model of an edentulous portion of the mandible requiring the replacement of teeth in the posterior region was developed using an available cone-beam computed tomography (CBCT) scan of a human. The CBCT scan was imported to CATIA V5 R20, and a solid mandibular model was created. The mandibular section had a height of 28 mm and a width of 16 mm. The width of cortical bone in the mandibular section was 1.2 mm (Figure 1).

 Three distinct types of IAC were chosen from commercially available implant systems, as follows:

- Implant 1: Tri-channel IAC (Nobel Biocare, Goteborg, Sweden)
- Implant 2: Conical IAC (Nobel Biocare, Goteborg, Sweden)
- Implant 3: Internal hex IAC (Norris)

All the implants were 10 mm in length and 4.3 mm in diameter.

 Corresponding abutments were selected to simulate platform-switched and platform-matched abutments, measurements of which were as follows.

###  Dimensions of abutment for platform-switched implants

1. Tri-channel implant: 3.5 × 5 mm
2. Conical implants: 3.6 × 5 mm
3. Internal hex connection: 3.8 × 6 mm

###  Dimensions of abutments for non-platform-switched implants

1. Tri-channel implant: 4.3 × 5 mm
2. Conical implants: 4.3 × 5 mm
3. Internal hex connection: 4.2 × 6 mm

**Figure 2:**
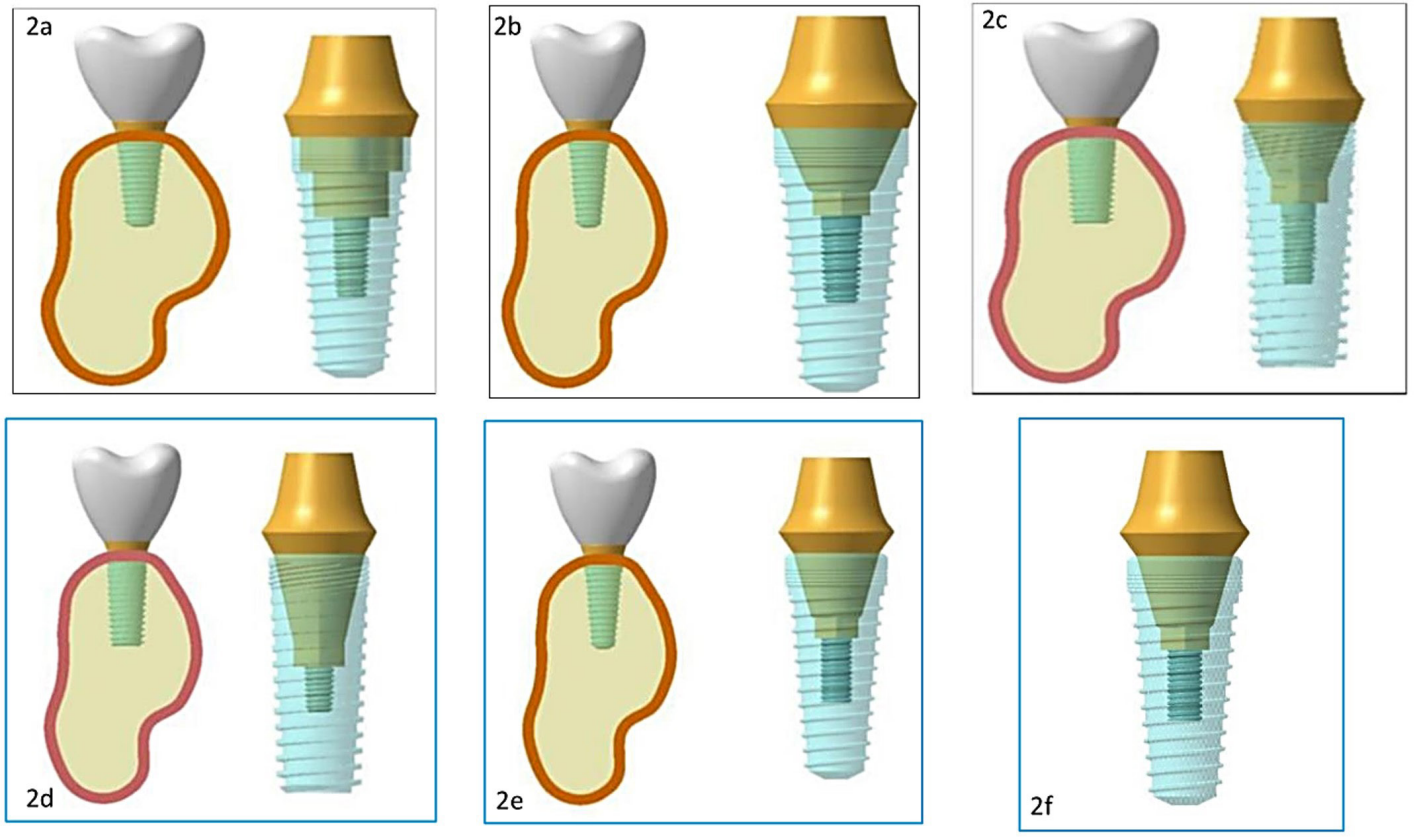
(2a) Platform-matched tri-channel implant abutment connection model. (2b) Platform-matched conical implant‒abutment connection model. (2c) Platform-matched internal-hex implant‒abutment connection model. (2d) Platform-switched tri-channel implant‒abutment connection model. (2e) Platform-switched conical implant‒abutment connection model. (2f) Platform-switched internal-hex implant‒abutment connection model

A total of 6 models were generated as follows:

 Model 1: A platform-matched dental implant with tri-channel IAC (Figure 2a)

 Model 2: A platform-matched dental implant with conical IAC (Figure 2b)

 Model 3: A platform-matched dental implant with internal hex IAC (Figure 2c)

 Model 4: A platform-switched dental implant with tri-channel IAC (Figure 2d)

 Model 5: A platform-switched dental implant with conical IAC (Figure 2e)

 Model 6: A platform-switched dental implant with internal hex IAC (Figure 2f).

###  Mesh creation

 Analysis of Systems (ANSYS) R15.0 software was used to mesh the models. The meshes of the implants and mandible models were created separately, and the separate meshes were combined to form six models with implants and the mandible. Tables 1, 2, and 3 present the total number of nodes and elements in structures.

**Table 1:** Total number of nodes and elements in structures

**Structure**	**Nodes**	**Element**
Cortical bone	33,260	12,007
Cancellous bone	61,242	37,274
Crown	6897	3908

**Table 2:** Total number of nodes and elements in platform-switched implants

	**Platform-switched implants**								
	**Tri-channel implant**			**Conical implant**			**Internal hex implant**		
Total nodes	1,93,860			2,05,535			1,85,006		
Total elements	1,03,875			1,09,222			93,635		
	**Implant**	**Abutment**	**Abutment screw**	**Implant**	**Abutment**	**Abutment screw**	**Implant**	**Abutment**	**Abutment screw**
Nodes	66,520	13,643	13,376	69,297	19,837	15,002	53,406	23,681	13,419
Element	38,866	4781	7539	40,561	6882	8590	30,848	7870	7576

**Table 3:** Total number of nodes and elements in non-platform-switched implants

	**Non-platform-switched implants**								
	**Tri-channel implant**			**Conical implant**			**Internal hex implant**		
Total nodes	1,91,679			1,98,454			1,85,879		
Total elements	1,03,128			1,06,492			9,55,843		
	**Implant**	**Abutment**	**Abutment screw**	**Implant**	**Abutment**	**Abutment screw**	**Implant**	**Abutment**	**Abutment screw**
Nodes	66,117	11,558	13,376	68,496	14,124	15,002	57,907	18,944	15,002
Element	38,595	4116	7539	40,199	4817	8590	33,632	6119	8590

###  Specifying material properties

Table 4 shows the attributes of the various materials used in the analysis.^12,13^ In terms of the mechanical characteristics of the simulated structures, the program made numerous assumptions.

**Table 4:** Specific materials properties

**Materials**	**Young’s modulus**	**Poisson's ratio**
Cortical bone	13,700	0.30
Cancellous bone	1370	0.30
Titanium implant	110,000	0.30
Crown	140000	0.28

1. Homogeneity: The mechanical characteristics of a material are assumed to be consistent throughout the structure.
2. Isotropy: Material characteristics are uniform in all directions.
3. Linear elasticity: The structure’s deformation or strain is proportional to the applied force and independent of the strain rate.

###  Bone‒implant interface conditions

 A continuous contact between bone and implant was assumed throughout the entire surface, resulting in no relative motion between the bone and implant under stress. The implant was assumed to be completely osseointegrated.

###  Load application

 For load application, three clinical scenarios were taken into consideration.^14^

1. Axial loads of 100-N magnitude were applied, which were directed downwards parallel to the long axis of the implant.
2. Non-axial loads of 100-N magnitude were applied at an angle of 30° from the long axis of the implant.
3. Combined load of 100-N magnitude, which included axial and non-axial loads.

###  Finite element analysis

 The processor of the FE software assessed these models, following which the post-processor displayed the results in the form of different color plots. Each color band denoted a different range of stress levels. The maximum von Mises stress was indicated in red, while the least was represented by blue. In the increasing sequence of stress distribution, the intermediate values were demarcated by bluish-green, green, yellow, and orange.^15^

## Results

 The results were displayed in the form of equivalent von Mises stress. The “equivalent stress of von Mises” is a value that gives an effective absolute magnitude of stresses, taking into consideration the principal stresses in three dimensions.^15^

 The models were evaluated for stress at the crestal bone, implant, and abutment screw using FEA. Tables 5, 6, and 7 present the stress values.

**Table 5:** Effect of implant‒abutment connection on the crestal bone

	**Tri-channel connection**			**Conical connection**			**Internal hex connection**		
	**Axial load**	**Non-axial load**	**Combined load**	**Axial load**	**Non-axial load**	**Combined load**	**Axial load**	**Non-axial load**	**Combined load**
Platform switched abutments	45	130	190	30	100	110	56	260	370
Non-platform-switched abutments	55	165	220	35	120	160	59	200	250

**Table 6:** Effect of implant‒abutment connection on the implant

	**Tri-channel connection**			**Conical connection**			**Internal hex connection**		
	**Axial load**	**Non-axial load**	**Combined load**	**Axial load**	**Non-axial load**	**Combined load**	**Axial load**	**Non-axial load**	**Combined load**
Platform-switched abutments	68	250	310	45	250	200	90	340	420
Non-platform-switched abutments	30	138	160	45	170	210	100	400	450

**Table 7:** Effect of implant-abutment connection on the abutment screw

	**Tri-channel connection**			**Conical connection**			**Internal hex connection**		
	**Axial load**	**Non-axial load**	**Combined load**	**Axial load**	**Non-axial load**	**Combined load**	**Axial load**	**Non-axial load**	**Combined load**
Platform-switched abutments	8	34.5	40	8	33	40	5.6	23	27
Non-platform-switched abutments	4.1	17	18	4.2	17	19	4.3	17.5	20

**Figure 3:**
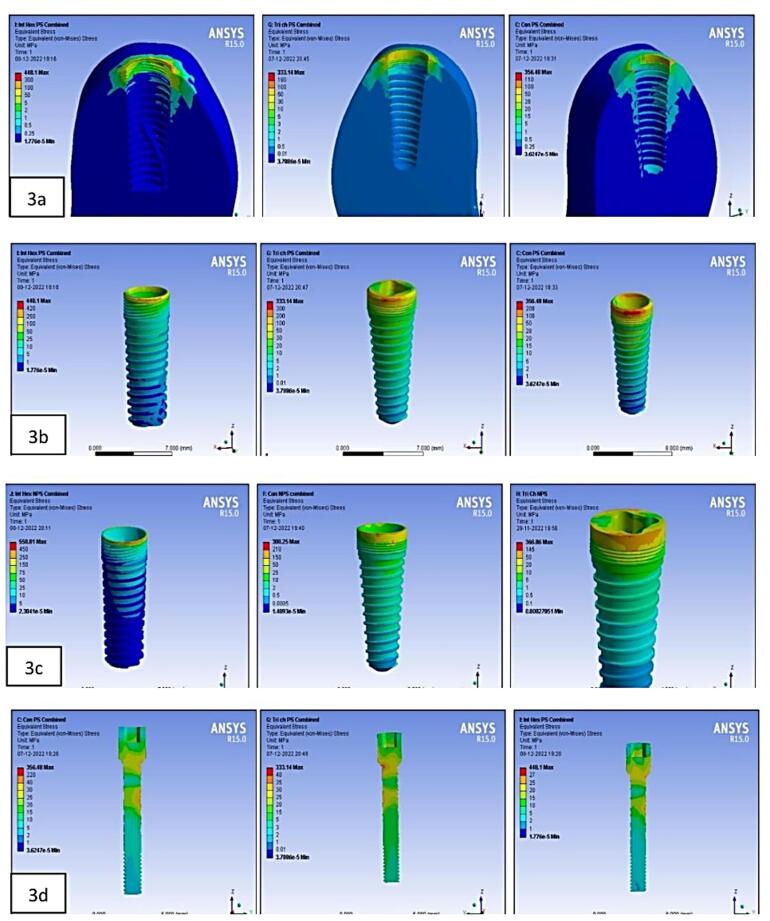
(3a) Stress at the crestal bone in platform-switched internal-hex implant on combined loading. Stress at the crestal bone in the platform-switched tri-channel implant on combined loading. Stress at the crestal bone in the platform-switched conical implant on combined loading. (3b) Stress on the implant in the platform-switched internal-hex implant on combined loading. Stress on the implant in the platform-switched tri-channel implant on combined loading. Stress on the implant in the platform-switched conical implant on combined loading. (3c) Stress on the implant in the non-platform-switched internal-hex implant on combined loading. Stress on the implant in the non-platform-switched conical implant on combined loading. Stress on the implant in non-platform-switched tri-channel implant combined loading. (3d) Stress on the abutment screw in the platform-switched conical implant on combined loading. Stress on the abutment screw in the platform-switched tri-channel implant on combined loading. Stress on the abutment in the platform-switched internal-hex implant on combined loading

**Figure 4:**
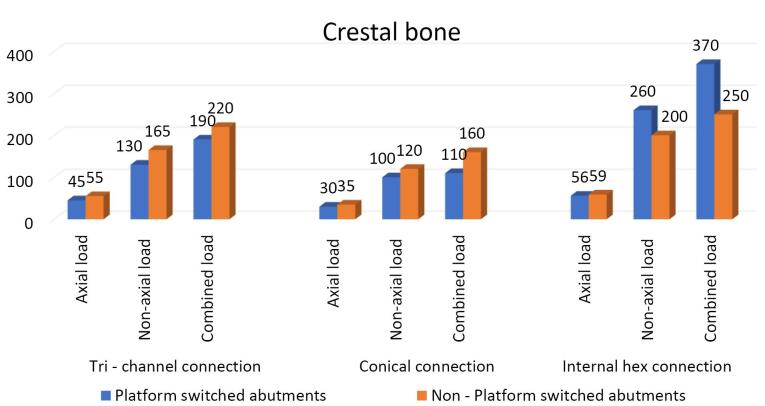
Effect of platform switching in three implant‒abutment connections on the crestal bone

**Figure 5:**
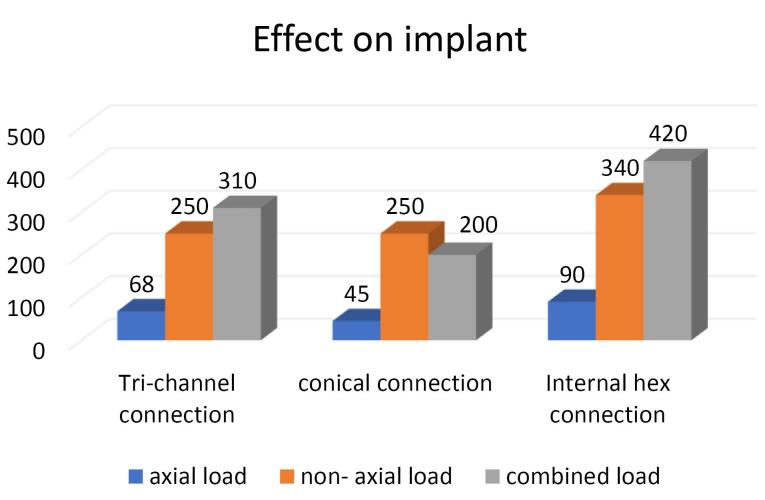
Effect of implant‒abutment connection in platform-switched implants on the implant

**Figure 6:**
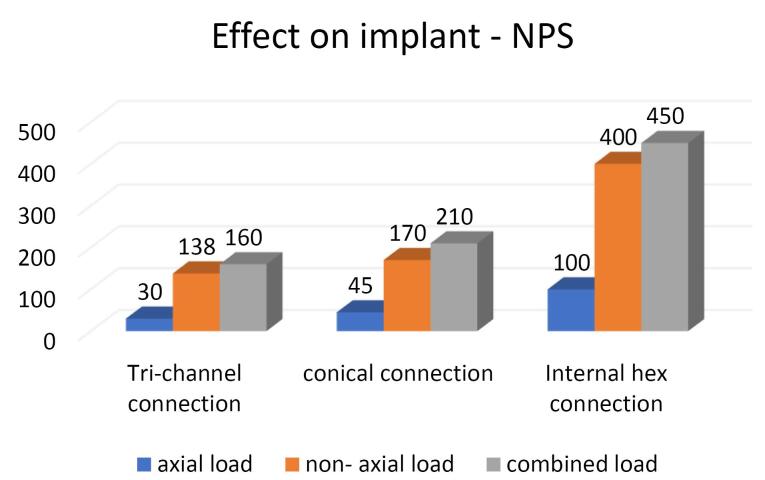
Effect of implant‒abutment connection in non-platform switched implants on the implant

**Figure 7:**
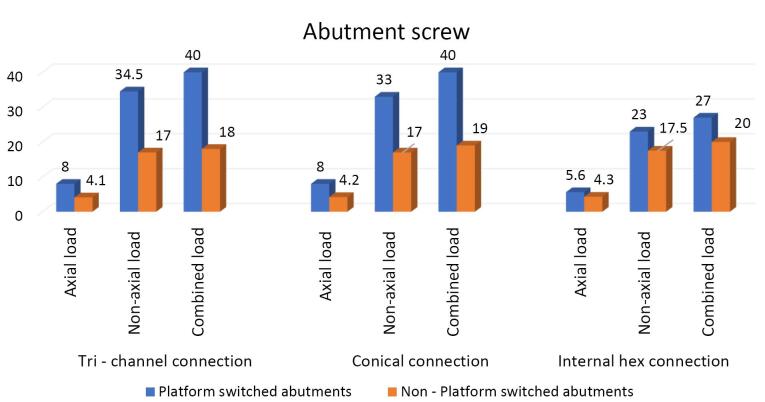
Effect of platform switching in three implant abutment connections on the abutment screw

 The following inferences were obtained from the results:

- On the crestal bone, the highest von Mises stresses were noticed in the internal hex implant abutment system, followed by the tri-channel implant abutment system and conical implant abutment system in platform-switched and non-platform-switched configurations (Figure 3a, Figure 4).
- On the implant, the highest von Mises stresses were observed in the internal hex implant abutment system, followed by the tri-channel implant abutment system and conical implant abutment system in the platform-switched configuration (Figure 3b, Figure 5).
- On the implant, the highest von Mises stresses were observed in the internal hex implant abutment system, followed by the conical implant abutment system and the tri-channel implant abutment system in the non-platform-switched configuration (Figure 3c, Figure 6).
- On the abutment screw, the highest von Mises stresses were observed in the conical implant abutment system, followed by the tri-channel implant abutment system and internal-hex implant abutment system in platform-switched and non-platform-switched configurations (Figure 3d, Figure 7).
- Compared to the non-platform-switched implants, platform-switched implants had a more favorable stress distribution on crestal bone (Figure 4).
- In contrast, platform-switched implants had higher abutment and IAC stresses than non-platform-switched implants (Figure 7).
- The von Mises stresses were higher for non-axial loading compared to axial loading.
- Similar stress levels were obtained in non-axial loads and combined loads in all three connections.

## Discussion

 The IAC is a critical and strategic area for implant longevity as it is responsible for biological and mechanical complications.^16^ Therefore, the implant’s material qualities and type of IAC must be considered for an implant assembly to endure biting pressures in patients.^17^

 Platform-switched connections can potentially prevent marginal bone loss by moving the IAC horizontally inwards.^18^ On histological evaluation of platform-switched implants, Cochran et al^19^ demonstrated that the implant‒abutment interface (micro-gap) is covered with connective tissue. Clinical studies have also demonstrated that less bone resorption occurs when a smaller diameter abutment is used with the platform switching technique.^20,21^

 Platform switching causes the inward shifting of the inflammatory cell infiltrate away from the crestal bone,^22^ and increases the distance between IAJ and the crestal bone level. Systematic reviews by Atieh et al,^23^ Strietzel et al,^24^ and Gupta et al^25^ stated that significantly less marginal bone loss was seen with platform-switched implants, concluding that platform switching helps preserve crestal bone around implants.

 Conducting in vivo trials to evaluate stress distribution on implant-supported prostheses is difficult because the dental implant components and the bone have exceedingly complicated geometry. Hence, FEA studies are carried out as they can replicate potential clinical situations.

 The normal biting force varies between 20 and 120 N^26^; thus, in our study, we used a static load of 100 N to simulate occlusal loading.^27,28^ The masticatory forces are oblique, with axial and lateral force components. Hence, in our study, we considered loads in three directions: axial, non-axial (at an angle of 30°), and combined load.

###  Effect of IAC on the crestal bone

 The results of the present study showed that the highest von Misses stresses in platform-switched implants were in internal hex connection, i.e., 370 MPa, followed by the tri-channel connection, i.e., 190 MPa, followed by the conical connection, i.e., 110 MPa. Similarly, in the non-platform-switched implants, the maximum von Misses stresses were seen in the internal hex connection, i.e., 250 MPa, followed by the tri-channel connection, i.e., 220 MPa, followed by the conical connection, i.e., 160 MPa. Our findings were consistent with those of Quaresma et al^29^ and Saidin et al.^30^

###  Effect of IAC on the Implant

 In the present study, the highest von Misses stresses in platform-switched implants were observed in the internal hex connection, i.e., 420 MPa, followed by the tri-channel connection, i.e., 310 MPa and conical connection with stress levels of 250 MPa. In the non-platform-switched implants, the highest stress was seen in the internal hex IAC, i.e., 450 MPa, followed by the conical connection, i.e., 210 MPa, followed by the tri-channel connection with stress levels of 160 MPa, which is consistent with the results of Quaresma et al^29^ and Saidin et al.^30^

 Kharsan et al^15^ reported that the stress levels were highest in the tri-channel IAC, followed by the conical-hex morse taper implant-abutment connection. These changes may be attributed to the changes in the nature of the forces used.

 Our analysis also found that the highest von Mises stresses were centered in the neck section of the implant‒abutment complex in all connections, consistent with studies by Takahashi et al^31^ and Akça et al^32^ (Figure 3b).

###  Effect on abutment screws 

**Figure 8:**
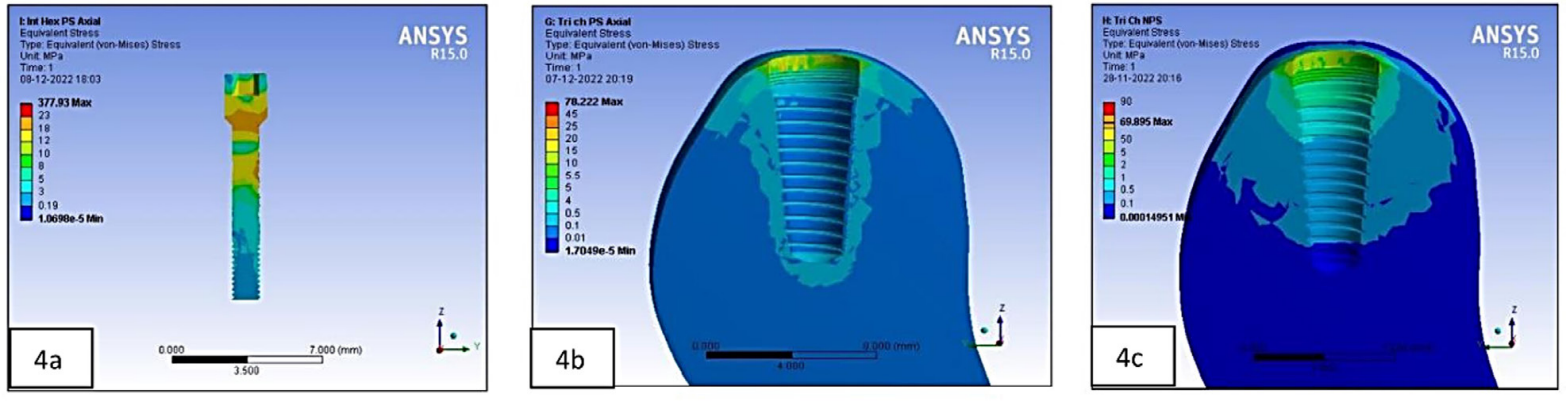
(8a) Stress on the abutment screw in the platform-switched internal-hex implant on non-axial loading. (8b) Stress at the crestal bone in the platform-switched tri-channel implant on axial loading. (8c) Stress at the crestal bone in the non-platform-switched tri-channel implant on axial loading

 In the present study, maximum von Misses stresses were detected at the center and the top of the abutment screw in all three connections (Figure 8a). Contradictory results were seen in a study by Takahashi et al,^31^ where the maximum stress concentration was observed at the bottom of the screw., possibly due to the difference in the design of the screw.

###  Effect of platform switching 

 Our results showed that platform-switched implants exerted less stress on the crestal bone than the platform-matched implants, except for the internal hex implant, which demonstrated higher values in platform-switched implants when combined loads were applied. The results of the present study are consistent with studies by Chang et al,^22^ Cimen et al,^33^ and Schrotenboer et al.^34^

 In platform-switched implants, the stresses on the alveolar bone were accumulated only in the area adjacent to the implant (Figure 8b). In contrast, in non-platform-switched implants, the stresses were dissipated over a larger area (Figure 8c).

 For the implant, a decrease in stresses was observed from the implant platform towards the apex in platform-matched models (Figure 8b), and platform-switched implants showed consistent stress levels throughout the implant fixture (Figure 8c). In the present study, the IAC, the abutment, and the abutment screw showed higher stresses in platform-switched implants than the non-platform-switched implants, consistent with Cimen et al.^33^

###  Effects of loading directions 

**Figure 9:**
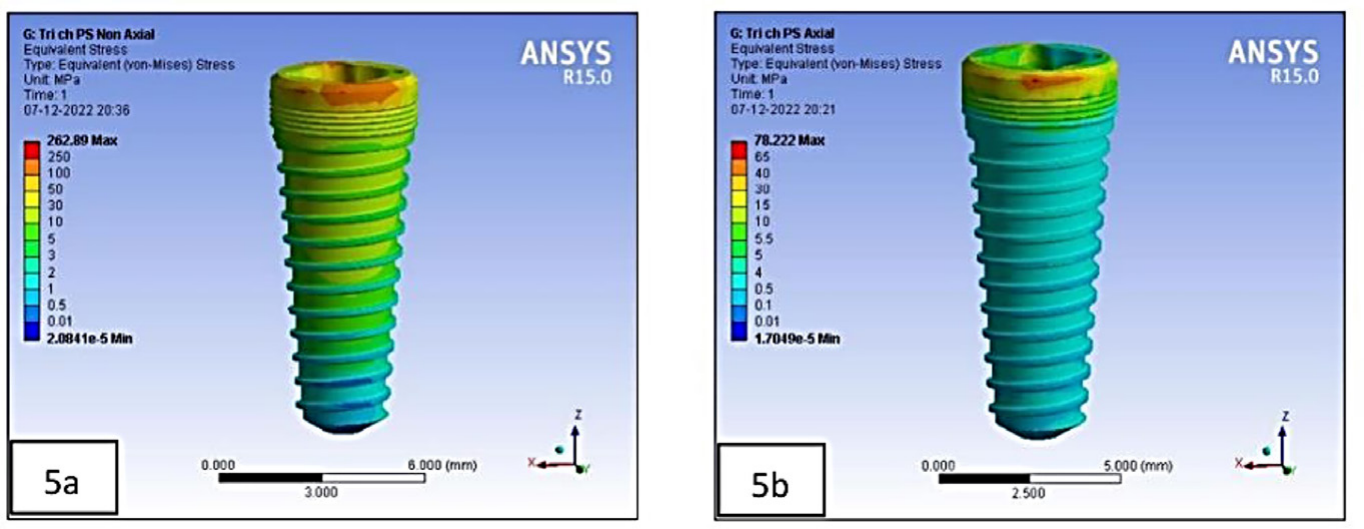
(9a) Stress on the implant in the platform-switched tri-channel implant on non-axial loading. (9b) Stress on the implant in platform-switched tri-channel implant on axial loading

 Vertical masticatory loads produce axial and non-axial forces that affect stress in the implant and bone.^3^ The highest von Mises stresses were seen in the combined direction, followed by the non-axial direction, and the least stresses were seen in the forces exerted in the axial direction (Tables 5, 6, and 7). However, all models had comparable stress distribution patterns when subjected to axial loading. These results are consistent with studies by Chun et al.^35^ A higher stress during oblique loading was predicted because the lateral forces during occlusion cause a bending moment within the prosthesis, prosthetic components, and supporting implants.^36^ Moreover, oblique loading causes changes in several stress concentration locations in implants. Higher stresses were also found to be localized in the apical area of the implant cylinders and abutments in non-axial-loaded models (Figure 9a) compared to the location in axially loaded models (Figure 9b), consistent with Takahashi et al.^31^

 Although the FEA is an accurate and exact tool for structural analysis, the current study has several shortcomings. It should be noted that the present simulations made various assumptions: (1) Although the implant was presumed to be completely osseointegrated, this may not be the case under clinical situations. (2) The cortical and the cancellous bone were considered linearly elastic, although a nonlinear assumption could be more suited for the jawbone simulation. (3) The static loads used in the study differ from the dynamic loading encountered during function.

 The IAJ is of primary importance to consider while selecting an implant system. Making the correct decisions on the IAC can enhance esthetics and longevity and provide for a structurally secure joint.

## Conclusion

 The implant abutment connection interface is a key feature in choosing an implant system. Making the correct decisions on the implant abutment connection interface can improve esthetics and longevity and provide for a structurally secure joint. This study’s findings indicated that implants with conical IAC with platform-switching configuration have more favorable stress distribution around the implant‒abutment interface compared to other implant‒abutment systems.

 On the crestal bone, the maximum von Mises stresses were observed in the internal hex implant abutment system, followed by the tri-channel implant abutment system and conical implant abutment system. On the implant and abutment screw, the maximum von Mises stresses were observed in the internal hex implant abutment system, followed by the conical implant abutment system and tri-channel implant abutment system. Platform-switched implants have more favorable stress distribution on crestal bone than non-platform-switched implants. However, further clinical research is suggested to establish it as a reliable method for implant selection.

## Competing Interests

 None.

## Ethical Approval

 Permission was granted by the Terna Dental College Ethics Committee with the number of TDC/EC/30/2020.

## Funding

 This research project was not funded by any external source.

## References

[1] Merickske-Stern R, Schroeder A, Sutter F, Krekler G. Oral Implantology: Basics, ITI Hollow Cylinder System. New York: Thieme Medical Publishers; 1996. p. 60-5.

[2] Brånemark PI, Zarb GA, Albrektsson T, Rosen HM. Tissue-Integrated Prostheses: Osseointegration in Clinical Dentistry. Chicago: Quintessence; 1985. p. 175-86.

[3] Geng JP, Tan KB, Liu GR (2001). Application of finite element analysis in implant dentistry: a review of the literature. J Prosthet Dent.

[4] Shadid R, Sadaqah N, Al-Omari W, Abu-Naba’a L (2013). Comparison between the butt-joint and morse taper implant-abutment connection: a literature review. J Implant Adv Clin Dent.

[5] Pessoa RS, Muraru L, Júnior EM, Vaz LG, Sloten JV, Duyck J (2010). Influence of implant connection type on the biomechanical environment of immediately placed implants - CT-based nonlinear, three-dimensional finite element analysis. Clin Implant Dent Relat Res.

[6] Nishioka RS, de Vasconcellos LG, de Melo Nishioka GN (2011). Comparative strain gauge analysis of external and internal hexagon, Morse taper, and influence of straight and offset implant configuration. Implant Dent.

[7] Chu CM, Huang HL, Hsu JT, Fuh LJ (2012). Influences of internal tapered abutment designs on bone stresses around a dental implant: three-dimensional finite element method with statistical evaluation. J Periodontol.

[8] Misch CE. ARABIC-Contemporary Implant Dentistry. Elsevier Health Sciences; 2007.

[9] Martini AP, Freitas AC Jr, Rocha EP, de Almeida EO, Anchieta RB, Kina S (2012). Straight and angulated abutments in platform switching: influence of loading on bone stress by three-dimensional finite element analysis. J Craniofac Surg.

[10] Rossi F, Zavanelli AC, Zavanelli RA (2011). Photoelastic comparison of single tooth implant-abutment bone of platform switching vs conventional implant designs. J Contemp Dent Pract.

[11] Weinstein AM, Klawitter JJ, Anand SC, Schuessler R (1976). Stress analysis of porous rooted dental implants. J Dent Res.

[12] Borchers L, Reichart P (1983). Three-dimensional stress distribution around a dental implant at different stages of interface development. J Dent Res.

[13] Daas M, Dubois G, Bonnet AS, Lipinski P, Rignon-Bret C (2008). A complete finite element model of a mandibular implant-retained overdenture with two implants: comparison between rigid and resilient attachment configurations. Med Eng Phys.

[14] Sarfaraz H, Paulose A, Shenoy KK, Hussain A (2015). A three-dimensional finite element analysis of a passive and friction fit implant abutment interface and the influence of occlusal table dimension on the stress distribution pattern on the implant and surrounding bone. J Indian Prosthodont Soc.

[15] Kharsan V, Bandgar V, Mirza A, Jagtiani K, Dhariwal N, Kore R (2019). Comparative evaluation of three abutment-implant interfaces on stress distribution in and around different implant systems: a finite element analysis. Contemp Clin Dent.

[16] Caricasulo R, Malchiodi L, Ghensi P, Fantozzi G, Cucchi A (2018). The influence of implant-abutment connection to peri-implant bone loss: a systematic review and meta-analysis. Clin Implant Dent Relat Res.

[17] Canullo L, Penarrocha-Oltra D, Soldini C, Mazzocco F, Penarrocha M, Covani U (2015). Microbiological assessment of the implant-abutment interface in different connections: cross-sectional study after 5 years of functional loading. Clin Oral Implants Res.

[18] Sasada Y, Cochran DL (2017). Implant-abutment connections: a review of biologic consequences and peri-implantitis implications. Int J Oral Maxillofac Implants.

[19] Cochran DL, Mau LP, Higginbottom FL, Wilson TG, Bosshardt DD, Schoolfield J (2013). Soft and hard tissue histologic dimensions around dental implants in the canine restored with smaller-diameter abutments: a paradigm shift in peri-implant biology. Int J Oral Maxillofac Implants.

[20] Zarandi A, Novin M (2017). Marginal bone loss around platform-switched and non-platform switched implants after two years of placement: a clinical trial. J Dent Res Dent Clin Dent Prospects.

[21] Lin HK, Lin JC, Pan YH, Salamanca E, Chang YT, Hsu YS (2022). Peri-implant marginal bone changes around dental implants with platform-switched and platform-matched abutments: a retrospective 5-year radiographic evaluation. J Pers Med.

[22] Chang CL, Chen CS, Hsu ML (2010). Biomechanical effect of platform switching in implant dentistry: a three-dimensional finite element analysis. Int J Oral Maxillofac Implants.

[23] Atieh MA, Ibrahim HM, Atieh AH (2010). Platform switching for marginal bone preservation around dental implants: a systematic review and meta-analysis. J Periodontol.

[24] Strietzel FP, Neumann K, Hertel M (2015). Impact of platform switching on marginal peri-implant bone-level changes A systematic review and meta-analysis. Clin Oral Implants Res.

[25] Gupta S, Sabharwal R, Nazeer J, Taneja L, Choudhury BK, Sahu S (2019). Platform switching technique and crestal bone loss around the dental implants: a systematic review. Ann Afr Med.

[26] Dittmer S, Dittmer MP, Kohorst P, Jendras M, Borchers L, Stiesch M (2011). Effect of implant-abutment connection design on load bearing capacity and failure mode of implants. J Prosthodont.

[27] Tabata LF, Rocha EP, Barão VA, Assunção WG (2011). Platform switching: biomechanical evaluation using three-dimensional finite element analysis. Int J Oral Maxillofac Implants.

[28] Natali AN, Pavan PG, Ruggero AL (2006). Analysis of bone-implant interaction phenomena by using a numerical approach. Clin Oral Implants Res.

[29] Quaresma SE, Cury PR, Sendyk WR, Sendyk C (2008). A finite element analysis of two different dental implants: stress distribution in the prosthesis, abutment, implant, and supporting bone. J Oral Implantol.

[30] Saidin S, Abdul Kadir MR, Sulaiman E, Abu Kasim NH (2012). Effects of different implant-abutment connections on micromotion and stress distribution: prediction of microgap formation. J Dent.

[31] Takahashi JM, Dayrell AC, Consani RL, de Arruda Nóbilo MA, Henriques GE, Mesquita MF (2015). Stress evaluation of implant-abutment connections under different loading conditions: a 3D finite element study. J Oral Implantol.

[32] Akça K, Cehreli MC, Iplikçioğlu H (2003). Evaluation of the mechanical characteristics of the implant-abutment complex of a reduced-diameter morse-taper implant A nonlinear finite element stress analysis. Clin Oral Implants Res.

[33] Cimen H, Yengin E (2012). Analyzing the effects of the platform-switching procedure on stresses in the bone and implant-abutment complex by 3-dimensional fem analysis. J Oral Implantol.

[34] Schrotenboer J, Tsao YP, Kinariwala V, Wang HL (2009). Effect of platform switching on implant crest bone stress: a finite element analysis. Implant Dent.

[35] Chun HJ, Shin HS, Han CH, Lee SH (2006). Influence of implant abutment type on stress distribution in bone under various loading conditions using finite element analysis. Int J Oral Maxillofac Implants.

[36] Hansson S (2000). Implant-abutment interface: biomechanical study of flat top versus conical. Clin Implant Dent Relat Res.

